# Cost analysis of venous stent reconstruction in the treatment of chronic lower extremity venous occlusive disease: Cost analysis of venous stent reconstruction for chronic lower extremity venous occlusive disease

**DOI:** 10.1186/s42155-026-00648-0

**Published:** 2026-09-23

**Authors:** Samir S. Jambhekar, Jacob J. Bundy, Spencer B. Lewis, Ravi N. Srinivasa, Anthony N. Hage, Eric J. Monroe, Jeffrey Forris Beecham Chick, David S. Shin, Matthew Abad-Santos, Mina S. Makary

**Affiliations:** 1 Division of Vascular and Interventional Radiology, Department of Radiology, The Ohio State University Wexner Medical Center, 395 W 12th Ave #460, Columbus, OH 43210 USA; 2 Community Radiology Associates 10461 Mill Run Circle, Suite 1020, Owings Mills, Baltimore, MD 21117 USA; 3 Division of Interventional Radiology, Department of Radiology, University of Washington, 1959 NE Pacific Street, Seattle, WA 98195 USA; 4 Division of Vascular and Interventional Radiology, Department of Radiology, University of California Los Angeles Health, 26585 Agoura Road, Suite 210, Calabasas, CA 91302 USA; 5 Division of Interventional Radiology, Department of Radiology, Thomas Jefferson University Hospitals, 111 South 11th Street, Philadelphia, PA 19107 USA; 6 Division of Interventional Radiology, Department of Radiology, University of Wisconsin, 600 Highland Ave, Madison, WI 53792 USA; 7 Division of Interventional Radiology, Department of Radiology, Keck School of Medicine of University of Southern California, 1500 San Pablo St, Los Angeles, CA 90033 USA; 8 Division of Vascular and Interventional Radiology, Department of Radiology, University of Southern California, Los Angeles, CA 90089 USA

## Introduction

As healthcare costs approach 20% of the United States’ gross domestic product, accurate healthcare cost accounting has become increasingly important [1]. Increases in the direct cost of hospital-based care have been predominant drivers of overall healthcare spending [1–3]. Furthermore, the emergence of value-based payment systems has bolstered the importance of precise, accurate cost estimates, ensuring fairness in reimbursements and identifying novel opportunities for cost reduction.

Time-driven activity-based costing (TDABC) is one such method for improving accuracy in medical cost accounting, having become a standard of process-based expense estimation in many industries [3–6]. TDABC examines the cost of a treatment modality across a longitudinal care cycle, encompassing all expenses necessary for treatment. To this end, process maps are prepared with principles borrowed from industrial engineering, while costs are tabulated through activity-based costing, a standard accounting technique [6]. The resources utilized and the associated cost rates are identified for each step in the process map. Cost is then estimated based on the product of each step’s duration and capacity cost rate [3, 4, 7]. Capacity cost rate is defined as the monetary cost of a resource per unit of time (in dollars per hour), calculated by dividing the total cost of a resource by the approximate time the resource is utilized [4]. This value may be calculated for all resources employed in a system, including human (as wages plus benefit costs divided by hours worked), equipment (as purchase cost divided by lifetime use), and occupancy (as rental costs divided by total annual productive occupancy time) [3, 4, 7, 8].

By examining the cost contributions of each resource independently, TDABC offers a more nuanced approach to cost drivers than charge-cost ratios and relative value unit (RVU) tabulation [3, 4]. TDABC not only highlights the costliest steps in a process but also facilitates the identification of the resources responsible for those costs while also employing a simple, linear model requiring only estimates of time and capacity cost rates as inputs [3].

This study reports the human and material cost drivers for endovenous iliocaval stent reconstruction for treating chronic venous occlusive disease (CVOD) using a TDABC technique. Process maps with cost components throughout the care episode were developed, including overhead, preoperative holding, procedure, and post-anesthesia care unit (PACU) care costs. The evaluated part of the care cycle included institutional overhead and patient care from preoperative holding, the procedure, and postoperative care in the PACU. Costs related to outpatient follow-up, medications, imaging, and management of complications were excluded. Accurate cost estimation may help identify cost savings opportunities and serve as a foundation for future comparative analyses between minimally invasive interventions and open surgical procedures.

## Materials and methods

### Study design

**Fig. 1:**
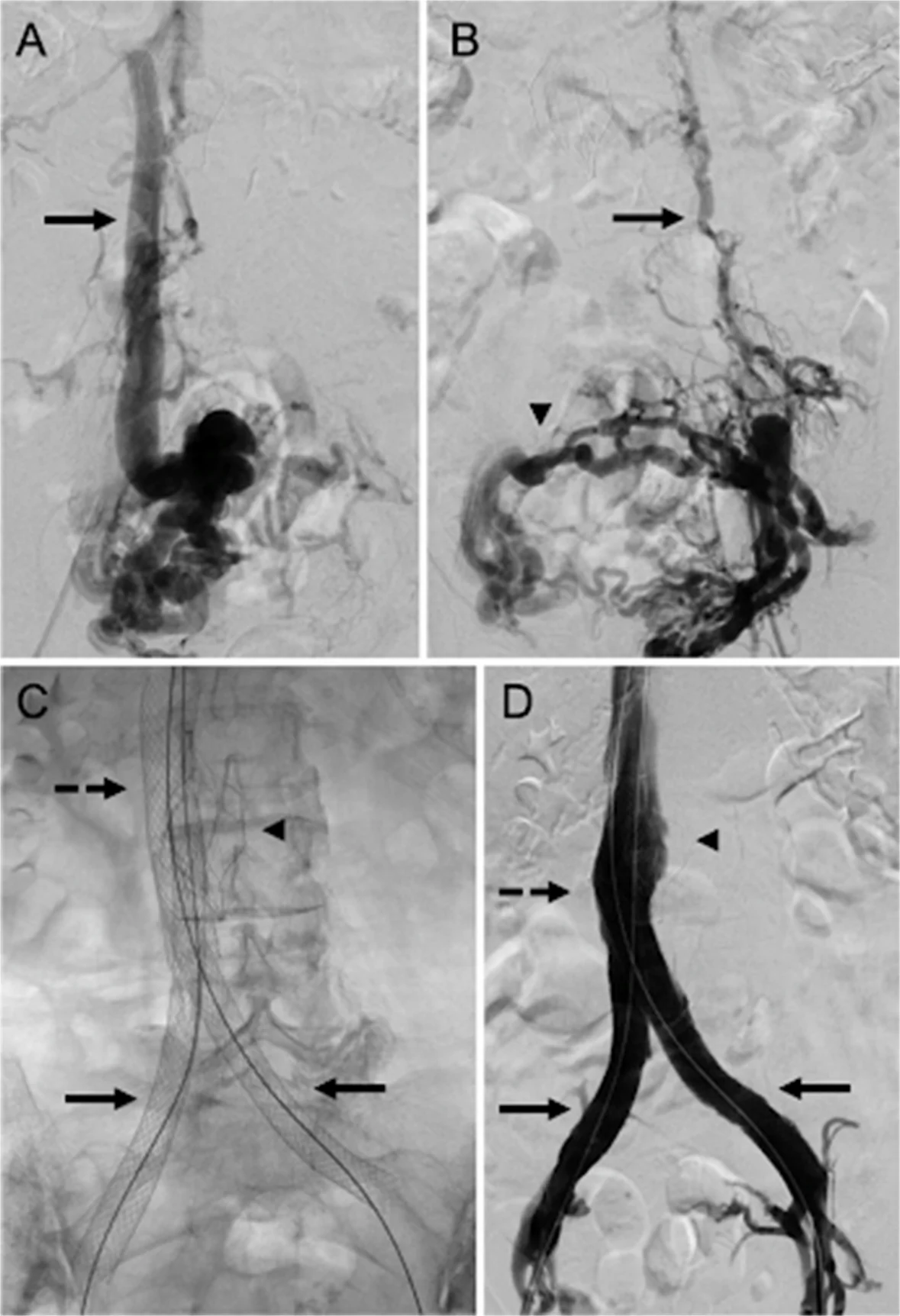
81-year-old female with TrapEase inferior vena cava filter-associated chronic bilateral iliocaval thrombosis. The patient presented with healed ulcerations of the left, greater than right, lower extremities and an inability to complete her one-mile daily walking routine due to lower extremity pain and fatigue. **A** Ascending venography, completed from a right common femoral vein approach, demonstrates complete chronic occlusion of the right common and external iliac veins. There was compensatory venous collateralization through the gonadal vein (solid black arrow). **B** Ascending venography, performed from a left common femoral vein approach, showed complete chronic occlusion of the left common and external iliac veins. Ascending lumbar (solid black arrow) and cross pelvic (black arrowhead) venous collaterals are seen. **C** Venous stent reconstruction was performed using a 22-mm Wallstent endoprosthesis within the inferior vena cava (dashed black arrow) and 16-mm “double barrel” common iliac venous Wallstents (solid black arrows). A decision was made to sequester, rather than remove, the TrapEase inferior vena cava filter (black arrowhead), given the patient’s age and potential trauma that may have occurred to the inferior vena cava during removal. **D** Completion of bilateral ascending iliocaval venography, performed after venous stent reconstruction, demonstrating brisk in-line flow through both iliac venous systems (solid black arrows) and inferior vena cava (dashed black arrow). The TrapEase inferior vena cava filter was sequestered (black arrowhead). The patient was able to resume her daily walking routine by one month. Healed ulcerations diminished by three months

*This was an Institutional Review Board-approved* and *Health Insurance Portability and Accountability Act-*compliant study*.* Thirty-one iliocaval venous stent reconstruction procedures for treating chronic iliocaval occlusive disease were reviewed. Chronic iliocaval occlusion was defined as symptomatic venous occlusive disease of > 4 weeks. Figure 1* demonstrates the restoration of flow in a patient with CVOD after ileocaval stent reconstruction.*

### Process map generation

**Fig. 2:**
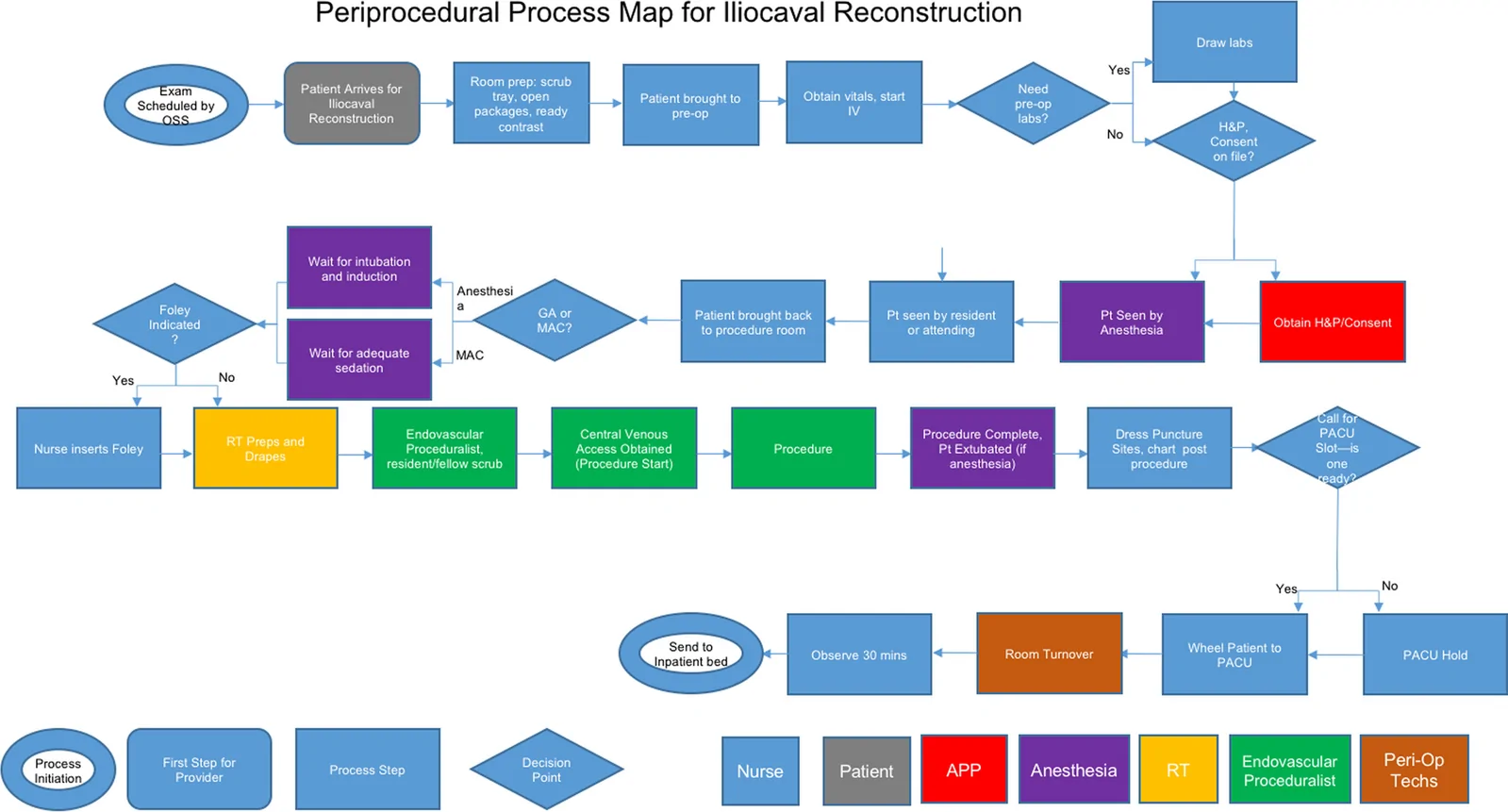
Process map showing all decision points, staff, and resources involved in iliocaval stent reconstruction. This analysis focuses on costs incurred between the time the patient enters the procedure room and the time the patient exits the procedure room

Model developments began with process maps for all parties involved in iliocaval stent reconstruction, created after interviews with representatives from each party. These process maps help define the care cycle by justifying time estimates, tying activities to costs in an easy-to-understand manner. *The process map for iliocaval stent reconstruction, demonstrating all steps, decision points, and individuals present or responsible for each step, is shown in *Fig. 2*.* Process duration for each step was estimated from electronic medical record evaluation (Epic; Verona, Wisconsin) or obtained from interviews with relevant parties. All costs are listed in United States dollars ($).

### Unit time estimation

The time estimates for iliocaval stent reconstruction were obtained from an electronic medical record evaluation. Event logs recorded the entry and exit of each team member, as well as the duration for which each resource was utilized. Intraprocedural and immediate periprocedural time were considered when measuring the duration of use. The mean of these times was used to obtain a representative duration for each step in the iliocaval stent reconstruction process map.

### Practical capacity and capacity cost rates

Practical capacity was defined as the actual time a resource, whether human or capital, was available [4]. Practical capacity was estimated using the assumption that most resources were used at 80–85% of their full capacity [5]. In consultation with the *Director of Clinical Operations*, practical capacity was estimated as 85%, based on the number of hours a typical employee works per day and the number of days that employee works per year, after accounting for leaves. Time estimates for each process-map step were obtained from electronic medical record (EMR) event logs and supplemented by structured staff interviews. Discrepancies between sources were resolved by prioritizing EMR timestamps when available. Times were averaged across multiple ileocaval stent reconstruction procedures to derive representative step durations. Practical capacity was set at 85% based on institutional staffing schedules and allowances for nonproductive time (e.g., breaks, administrative tasks). This assumption was applied uniformly to both personnel and room resources to simplify analysis, as precise utilization rates for individual in-room resources were not available and were unlikely to reflect full efficiency. Capacity cost rates were estimated for all relevant resources by dividing annual salary estimates by practical capacity time (in hours per year). Cost inputs were primarily derived from institutional and publicly available data from 2017–2018 and are reported in nominal U.S. dollars without inflation adjustment.

### Staff practical capacity costs

Salaries and hourly wages, including employee benefits, were calculated from payroll records, the *United States Bureau of Labor Statistics Occupational Employment Statistics* database, and other publicly available institutional data [9–13].

### Capital equipment

Anesthesia machines, fluoroscopy tables, and other fixed components of the interventional suite were defined as capital equipment, which was defined as non-disposable materials purchased by the institution for long-term use. Capital equipment costs were obtained from the *Director of Clinical Operations*. The total equipment cost of an interventional suite without computed tomography capability was $4.5 million, with a seven-year depreciation timeline and a ten-year replacement time horizon. Equipment was assigned a value of $0 at the end of its lifespan. Based on historical data, the budgeted capacity of the interventional suites was approximately 948 h per week for 12 rooms or 79 h per week per room. Assuming a practical capacity equal to 85% of full capacity, this yielded a cost rate of $129 per room per hour. Non-disposable multipurpose products and fixed angiography suite material (such as hospital beds, ultrasound, angiography tables, and electronic medical records) were not included in the analysis due to multiple confounding uses of these items and relatively small per-patient impacts when factoring in depreciation. The proprietary nature of institutional equipment costs precludes a formal capital analysis.

### Disposable material costs

Unit prices were obtained from the institution’s charge master and compared to data obtained through market analysis and published data to obtain cost estimates [14, 15]. Materials selected for sensitivity analysis included guidewires, catheters, stents, balloons, and sheaths.

### Cost sensitivity analyses

*The parameters for the base case analysis are summarized in *Table 1*.* Following the development of the base case, univariate sensitivity analyses were performed to determine the cost contribution of each variable [16]. Two classes of variables were analyzed: human labor costs and disposable materials costs. *The range and rationale for the values studied are summarized in *Table 2*.* The lower and upper bounds for salary were the 10th and 90th percentiles of national data from the *Bureau of Labor Statistics* occupational employment statistics database or other publicly available databases [9–11, 14]. Task time varied from 50–200% of the mean procedure duration, which was retrospectively obtained through institutional data. Disposable material cost variations were estimated using published online market values, discussions with vendors, and the institutional central supply department.

**Table 1:** Base case cost model, per case, for iliocaval stent reconstruction. The table presents model variables grouped by labor costs and equipment costs. Associated data sources are provided for reference

**Human staffing costs**	
**Interventional Radiology (IR) nurse**	
Adjusted hourly wage ($/hr)	$51.86
Time required/case (hr)	9.094
Total cost/case	$471.58
**IR tech**	
Adjusted hourly wage	$43.52
Time required/case (hr)	14.375
Total cost/case	$625.64
**Fellow**	
Adjusted hourly wage	$36.24
Time required/case (hr)	6.24
Total cost/case	$226.14
**IR attending**	
Adjusted hourly wage	$196.41
Time required/case (hr)	6.24
Total cost/case	$1,225.59
**Anesthesiologist**	
Adjusted hourly wage	$218.33
Time required/case (hr)	4.86
Total cost/case	$1,061.11
**Certified nurse anesthetist**	
Adjusted hourly wage	$121.42
Time required/case (hr)	6.37
Total cost/case	$773.42
Total Base Case Wage Cost	$4,383.48
**Disposable materials costs**	
**Balloon catheter**	
Base Cost	$438.09
Mean Quantity	11.5
**Microcatheter**	
Base Cost	$496.00
Mean Quantity	5.5
**Guidewire**	
Base Cost	$49.00
Mean Quantity	11
**Sheath**	
Base Cost	$50.00
Mean Quantity	7.75
**Stent**	
Base Cost	$1,250.00
Mean Quantity	10.5
**Total base case disposable cost**	$21,817.54
**Total base case cost**	$26,201.01

**Table 2:** Univariate sensitivity analysis range of values and total costs. The table presents the minimum and maximum values over which each variable was investigated. The rationale for the range is provided for reference. Total cost refers to the model output cost with all other variables held static to the base case. The base case cost is $26,201.01 per case

**Human staffing costs**	Value	Total Cost of Iliocaval Stent Reconstruction
Interventional Radiology (IR) nurse hourly wage		
Minimum	$35.70	$26,054.05
Maximum	$78.02	$26,438.91
IR nurse time required/case		
Minimum	7.15	$26,100.19
Maximum	11.5	$26,325.78
IR Tech hourly wage ($/hr)		
Minimum	$29.29	$25,995.57
Maximum	$62.57	$26,476.05
IR tech time required/case		
Minimum	8	$25,920.85
Maximum	18.2	$26,364.76
Fellow hourly wage ($/hr)		
Minimum	$28.81	$26,154.64
Maximum	$39.79	$26,223.17
Fellow time required/case		
Minimum	5.75	$26,183.19
Maximum	7.15	$26,233.93
IR attending hourly wage ($/hr)		
Minimum	$72.73	$26,118.31
Maximum	$246.97	$26,339.51
IR attending time required/case		
Minimum	5.75	$26,133.28
Maximum	7.15	$26,422.22
Anesthesiologist hourly wage ($/hr)		
Minimum	$156.57	$25,990.55
Maximum	$272.22	$26,384.61
Anesthesiologist time required/case		
Minimum	0.3	$25,533.71
Maximum	9.43	$26,551.89
Certified Nurse Anesthetist hourly wage ($/hr)		
Minimum	$81.79	$25,948.54
Maximum	$157.58	$26,431.38
CRNA time required/case		
Minimum	5.75	$26,125.63
Maximum	7.15	$26,295.62
**Disposable materials costs**		
Guidewire cost (per unit)		
Minimum	$19.00	$25,871.01
Maximum	$560.00	$31,822.01
Guidewire quantity		
Minimum	6	$25,956.01
Maximum	15	$26,397.01
Balloon Catheter Cost (per unit)		
Minimum	$193.14	$23,384.09
Maximum	$970.39	$32,322.46
Balloon Catheter Quantity		
Minimum	9	$25,105.79
Maximum	13	$26,858.15
Microcatheter cost (per unit)		
Minimum	$375.00	$25,535.51
Maximum	$1,070.00	$29,358.01
Microcatheter quantity		
Minimum	3	$24,961.01
Maximum	8	$27,441.01
Sheath Cost (per unit)		
Minimum	$25.00	$26,007.26
Maximum	$150.00	$26,976.01
Sheath Quantity		
Minimum	5	$26,063.51
Maximum	12	$26,413.51
Stent Cost (per unit)		
Minimum	$450.00	$17,801.01
Maximum	$2,070.00	$34,811.01
Stent Quantity		
Minimum	7	$19,326.01
Maximum	13	$29,326.01
**Multivariate cost analysis**		
Minimum		$7,842.95
Maximum		$66,066.05

Institutional salary data and materials costs are proprietary and not individually reported, but are incorporated into an overall cost comparison as a multivariate secondary analysis. Additional multivariate sensitivity analyses were performed by varying the minimum and maximum values for all model variables to generate a base case cost range. The cost range of additional stents, which was not utilized in the base case, was determined by adding supplemental agent costs and quantities to base case values in a multivariate fashion.

## Results

### Iliocaval stent reconstruction costs

Thirty-one iliocaval venous stent reconstruction procedures for the treatment of chronic iliocaval occlusive disease were analyzed. The mean procedure duration in the angiography suite for iliocaval stent reconstruction was 6.24 h (range: 5.75–7.15 h). The base cost of an iliocaval stent reconstruction was $26,201.01 (Table 1). Institution-specific capital equipment costs increased total costs by 3.1%. Multivariate sensitivity analyses, with all minimum and maximum values for cost input variables, yielded a cost range of $7,842.95 (minimum) to $66,066.05 (maximum). This variance was largely attributable to the variable use of the human staffing and disposable materials seen on Table 1. Using local salary information and negotiated prices for materials as parameters, the true cost per case of iliocaval stent reconstruction was $25,750.33.

### Human staffing costs

**Fig. 3:**
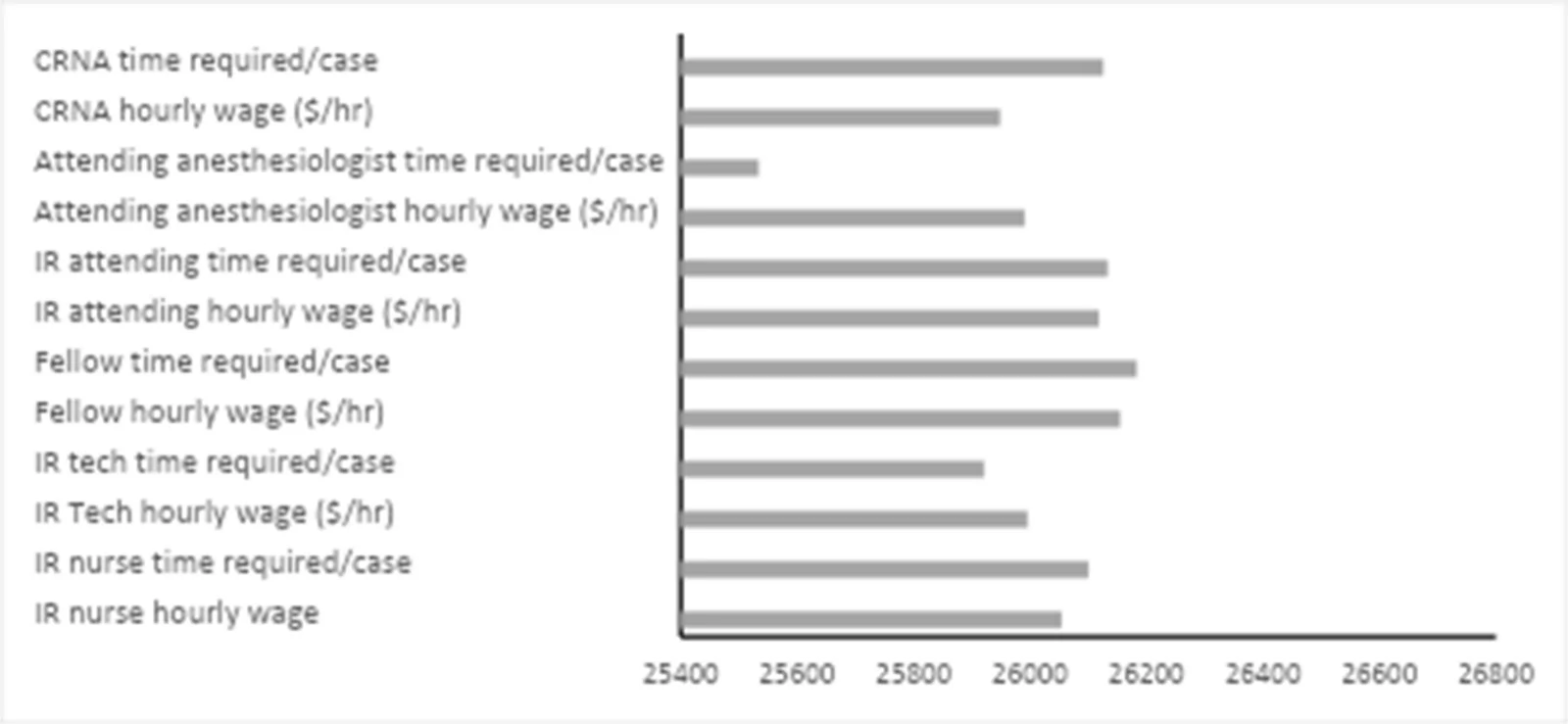
Univariate sensitivity analysis of human labor factors related to the cost of iliocaval stent reconstruction per case. The central vertical line indicates the base case and corresponds to a cost of $26,201.01. Each variable is modeled as an uncertain variable, from minimum to maximum studied values around the base case value, with all other variables held static. Gray bars to the left of the vertical line represent the degree of total cost reduction, and the striped bars to the right of the vertical line represent the degree of total cost increase

*Univariate sensitivity analyses of labor factors related to iliocaval stent reconstruction are summarized in *Table 2* and *Fig. 3. Per univariate sensitivity analysis, the largest factor contributing to labor cost was the time an anesthesiologist was required to be present for the procedure (range: $25,533.71-$26,551.89), followed by nurse anesthetist wages (range: $25,984.54-$26,431.38), interventional radiology (IR) technologist wages (range: $25,995.57-$26,475.05), and technologist-hours per case (range: $25,920.85-$26,364.76).

### Disposable materials costs

**Fig. 4:**
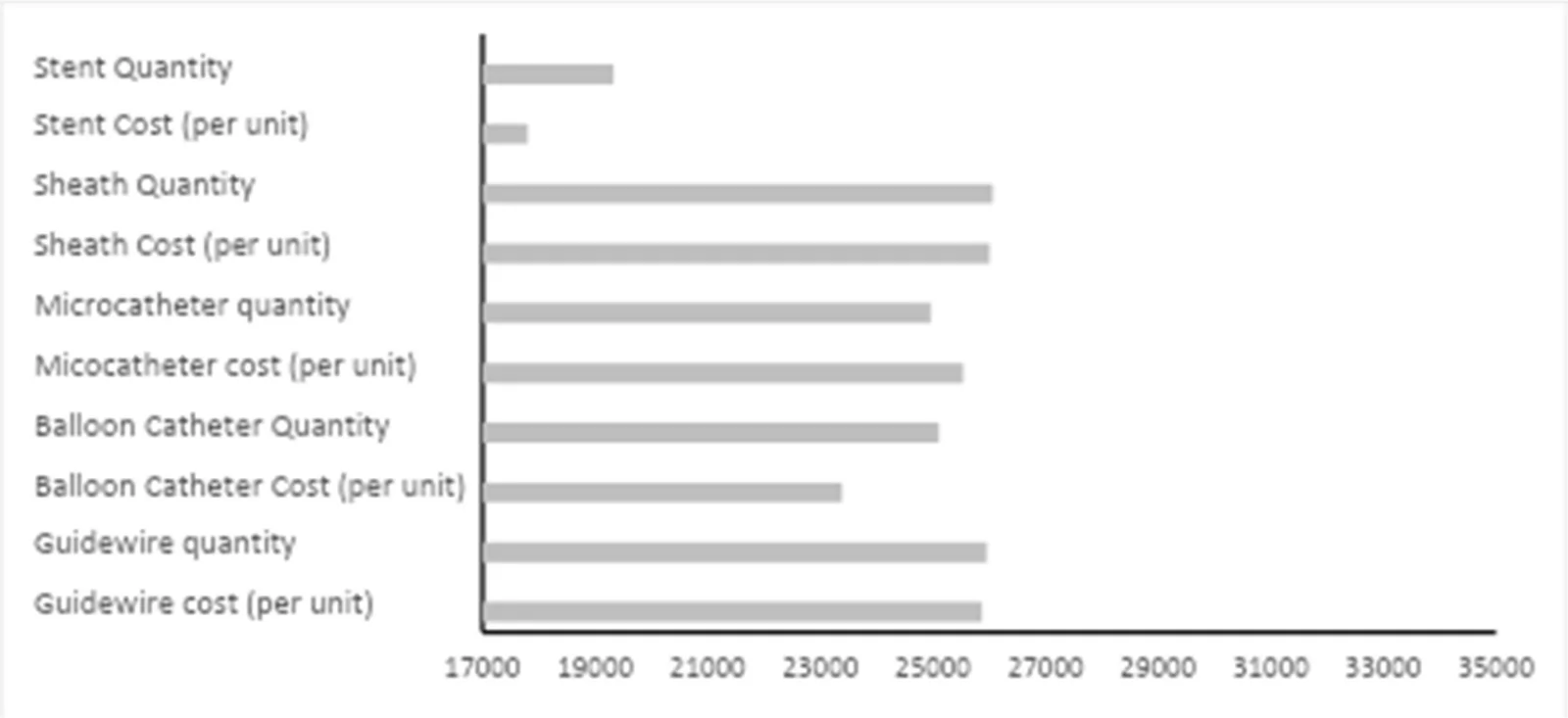
Univariate sensitivity analysis of material factors related to the cost of iliocaval stent reconstruction per case. The central vertical line indicates the base case and corresponds to a cost of $26,201.01. Each variable is modeled as an uncertain variable, from minimum to maximum studied values around the base case value, with all other variables held static. Gray bars to the left of the vertical line represent the degree of total cost reduction, and the striped bars to the right of the vertical line represent the degree of total cost increase

*Univariate sensitivity analyses of disposable material costs are summarized in *Table 2* and *Fig. 4. By associating each disposable material item in a procedure with a cost, one can quickly appreciate how complex cases involving the use of multiple items can lead to a wide variance in procedure costs. This figure also illustrates how disposable materials had a substantially greater impact on costs than wages. Venous stent price had the greatest impact on iliocaval stent reconstruction costs (range: $17,801.01-$34,811.01). The number of stents used also had a substantial effect on costs (range: $19,326.01-$29,363.01), as did the cost of balloon catheters (range: $23,384.09-$32,322.46).

## Discussion

TDABC is a versatile methodology that has been used to identify cost drivers in various medical settings, including neurosurgery, otorhinolaryngology, and radiology [15–17]. In many contexts studied, staffing costs dominate expenses. This study, however, demonstrated that disposable material costs were almost an order of magnitude more substantial (range: $7,842.95-$66,066.05). Substantial variability in both device type and quantity was observed for iliocaval stent reconstruction, including guidewires, catheters, sheaths, balloons, venous stents, thrombectomy devices, and recanalization tools. The lower bound of the multivariate sensitivity analysis reflects scenarios with minimal device utilization and lower-end unit cost assumptions, whereas the upper bound reflects complex cases requiring multiple high-cost disposable materials modeled at their upper price ranges, such as requiring recanalization with multiple stents. Identifying the costliest steps during iliocaval stent reconstruction and the disposable materials responsible for the most cost variability may allow for cost reductions during future interventions.

This investigation found that disposable materials accounted for 83.2% of the base cost of an iliocaval stent reconstruction, whereas personnel costs accounted for 16.8%. Similar rates were found during a retrospective calculation for operational expenses in conventional transarterial chemoembolization, in which disposable materials accounted for 62% of costs. In comparison, personnel and patient recovery costs contributed 25% and 13%, respectively [18]. TDABC analysis of arteriovenous malformation and venous malformation found that material costs accounted for 45% and 75% of total costs, while personnel costs accounted for 13–21%. Arteriovenous malformation treatment can be prolonged and costly. Other TDABC studies of IR procedures also found that disposable materials comprised a majority of procedure costs. A retrospective analysis to characterize and compare costs of transarterial chemoembolization, transarterial radioembolization, and ablation found that consumables contributed to 58%, 90%, and 65% of the costs of these procedures, respectively [19]. A retrospective TDABC study of uterine artery embolization found that consumables accounted for 51% of total cost [20]. In contrast, venous malformation treatments are often rapid and inexpensive, suggesting the cost savings achieved through TDABC in this study may be generalizable across IR procedures [21]. The change in cost contributions from traditional labor and capital investments to disposable materials has an interesting impact on IR. Due to its constant evolution, IR has seen increasingly sophisticated devices that have helped facilitate more accurate deployment of therapies. While technological advancements and innovations improve procedural successes and patient outcomes, they generally increase costs.

In this analysis, venous stents were the disposable materials with the greatest costs and the most variability. Proper venous stent number, size, and placement are vital to prevent stent migration and improve patency rates. A relative lack of stents designed for the iliocaval venous anatomy likely contributes to an increased number of stents needed for the modular creation of stent constructs of varying caliber and branching. Meanwhile, meticulous anatomic mapping before stent selection reduces unintentional waste of stents during iliocaval stent reconstruction. The adoption of intraprocedural intravascular ultrasound (IVUS) is one planning technique to optimize venous stent selection (number, diameter) and placement. IVUS is currently used in 73% of iliocaval stent reconstructions. Despite its upfront costs, IVUS may facilitate proper stent number and sizing, reducing waste [22, 23].

Regarding personnel costs, the present study found that the largest contributing component was the time an anesthesiologist was required to be present. General anesthesia is generally appropriate for the recanalization of chronic iliocaval thrombosis because angioplasty of stenosed and occluded venous segments may be painful [24]. Opportunities for conscious sedation in select patients may reduce the utilization of anesthesiologists and reduce costs. TDABC-driven methods can further reduce costs by reducing idle time during IR procedures [25]. These changes involve upfront and ongoing investments, such as assigning more nurses to preoperative areas, streamlining the preoperative process, and expanding post-anesthesia capacity. TDABC may be used to determine the optimal setting for particular procedures, including venous reconstruction. Inferior vena cava filter retrieval is done in both IR and surgical settings. A study at a tertiary care center found that patients in surgical settings experienced significantly higher postprocedural complication rates and incurred higher costs. IR retrievals had higher technical success rates and shorter fluoroscopy times [26]. In recent years, minimally invasive catheter-directed therapies have seen significant growth in their use for treating common venous conditions in Medicare beneficiaries, such as deep vein thromboses. Despite associated costs, IR procedures to relieve venous outflow occlusive disease have shown significant clinical benefit. A systematic review and meta-analysis of studies with treatment of nonthrombotic and chronic post-thrombotic syndrome patients reported complete symptom relief at the final follow-up visit was reported in 69% to 82% of patients for pain, 64% to 68% of patients for edema, and 71% to 81% of patients for ulcer healing [27]. As healthcare funding comes under greater strain, TDABC could be used to optimize protocols and control costs in various similar procedures in order to improve access to IR procedures [28].

There are limitations to this study. The analysis was retrospective and limited to a single institution. Costs may vary with geographic location, inflation, internal cost accounting methodology, and local treatment preferences. While the study measured time by the duration staff were in the procedural room and other costs incurred from preoperative holding, the procedure itself, and patient care in the post-anesthesia care unit, post-procedural management, including clinical follow-up, medical management, and imaging, was not assessed. Many time estimations for procedure steps came from a chart review of procedure logs with timeline discrepancies. Finally, while costs were evaluated as independent variables, they are likely confounded. Complex reconstructions likely involved greater disposable material costs and prolonged procedure times with inherent increases in personnel costs. Performance of endovascular stent reconstruction for chronic iliocaval disease at specialized, high-volume centers of excellence could reduce disposable material and personnel costs through more efficient procedures. However, this analysis did not study the effects of operator experience on procedural costs.

Healthcare organizations have successfully used TDABC to reduce the total number of full-time equivalents to staff by 17% and reduce the cost per patient of providing care by 46% [29]. The breadth of procedures in IR and its rapidly changing nature suggest it may benefit from a structured approach in applying TDABC [19]. The unique position of IR necessitates increased consideration regarding the significant costs of consumables, in addition to personnel costs, to enhance the value for patients and limit expenditures.

## Conclusion

Human labor and disposable material costs, namely anesthesia staffing costs and stent and catheter selection, are large, potentially modifiable drivers of the overall cost of iliocaval stent reconstruction for CVOD. Future studies are necessary to define an iliocaval venous reconstruction protocol that delivers cost savings without compromising outcomes.

## Data Availability

All data generated or analyzed during this study are included in this published article [and its supplementary information files].

## Abbreviations

TDABC: Time-driven activity based costing
RVU: Relative value unit
CVOD: Chronic venous occlusive disease
PACU: Post-anesthesia care unit
EMR: Electronic medical record
IR: Interventional radiology
IVUS: Intravascular ultrasound
